# Frequency of* BCR-ABL* Transcript Types in Syrian CML Patients

**DOI:** 10.1155/2016/8420853

**Published:** 2016-05-30

**Authors:** Sulaf Farhat-Maghribi, Wafa Habbal, Fawza Monem

**Affiliations:** ^1^Department of Biochemistry and Microbiology, Faculty of Pharmacy, Damascus University, Damascus, Syria; ^2^Clinical Laboratories Department, Al-Assad Hospital, Damascus University, P.O. Box 10769, Damascus, Syria

## Abstract

*Background*. In Syria, CML patients are started on tyrosine kinase inhibitors (TKIs) and monitored until complete molecular response is achieved.* BCR-ABL* mRNA transcript type is not routinely identified, contrary to the recommendations. In this study we aimed to identify the frequency of different* BCR-ABL* transcripts in Syrian CML patients and highlight their significance on monitoring and treatment protocols.* Methods*. CML patients positive for* BCR-ABL* transcripts by quantitative RT-PCR were enrolled.* BCR-ABL* transcript types were investigated using a home-made PCR method that was adapted from published protocols and optimized. The transcript types were then confirmed using a commercially available research kit.* Results*. Twenty-four transcripts were found in 21 patients. The most common was b2a2, followed by b3a2, b3a3, and e1a3 present solely in 12 (57.1%), 3 (14.3%), 2 (9.5%), and 1 (4.8%), respectively. Three samples (14.3%) contained dual transcripts. While b3a2 transcript was apparently associated with warning molecular response to imatinib treatment, b2a2, b3a3, and e1a3 transcripts collectively proved otherwise (*P* = 0.047).* Conclusion*. It might be advisable to identify the* BCR-ABL* transcript type in CML patients at diagnosis, using an empirically verified method, in order to link the detected transcript with the clinical findings, possible resistance to treatment, and appropriate monitoring methods.

## 1. Introduction

The Philadelphia (Ph) chromosome (derivative chromosome 22), resulting from a translocation between chromosomes 9 and 22, was the first disease-specific chromosomal abnormality to be associated with a malignancy, namely, chronic myelogenous leukemia (CML) [1]. Advancements in cytogenetic and molecular methods have led to the identification of the genes involved in the t(9; 22) breakpoints,* ABL1* and* BCR*, respectively. The joining of the two genes results in a* BCR-ABL* fusion oncogene transcribed into a chimeric messenger RNA (mRNA) [2, 3]. This mRNA is translated into a chimeric protein with a constitutive tyrosine kinase activity that activates cell cycle related pathways and induces the malignant proliferation of the chronic phase of CML. Tyrosine kinase inhibitors (TKIs) were rationally designed to target this fusion protein and specifically block its enzymatic action, leading to a high frequency of remission and better survival rates in CML patients [4, 5].

Due to this hierarchy of cause and effect, the structure of the chimeric* BCR-ABL* mRNA will differ according to the breakpoint in the corresponding genes and subsequently so will the structure of the resulting protein. The breakpoint within the* ABL1* gene is almost always at the second exon (a2), while the breakpoint in the* BCR* gene varies between the different patients and malignancies and can be localized to one of three regions, major* BCR* (M-*bcr*), minor* BCR* (m-*bcr*), and micro* BCR* (*μ*-*bcr*) [3]. In the majority of CML cases, the* BCR-ABL* fusion junction contains a breakpoint in the M-*bcr* region at exon e13 (b2) or exon e14 (b3) and the oncogene is translated into one of two 210-kDa proteins (p210^*BCR-ABL*^) differing by 25 amino acids depending on the exons included. Both e13 and e14 fusion junctions could be seen in the same patient usually due to alternative splicing. Some CML patients have* BCR-ABL* junctions containing breakpoints in the m-*bcr* region at exon e1 and the oncogene is translated into a 190-kDa protein (p190^*BCR-ABL*^). Rarely, patients present with a* BCR-ABL* oncogene containing a breakpoint within the *μ*-*bcr* region at exon e19 that produces a 230-kDa tyrosine kinase (p230^*BCR*-*ABL*^) [6].

Hence, the* BCR-ABL* fusion gene and its corresponding mRNA transcripts and protein forms have been the subject of several studies and significant differences were found between patients with the b2a2, b3a2, rarer transcripts, or a combination of two or more transcripts regarding the clinical aspects and progression of the leukemia as well as response to treatment [7–11]. Populations also showed different percentages of the two most common transcripts b2a2 and b3a2, and of the rarer transcripts in their CML patients [12–24], noting that patients with rare transcripts represent another challenge at the level of molecular diagnosis and monitoring since those transcripts may be undetectable by quantitative reverse transcription polymerase chain reaction (qRT-PCR) monitoring assays, consequently producing false-negative results [25].

In Syria, chromosome banding is performed at diagnosis of CML patients to confirm their Ph+ status; they are started on first line TKI imatinib mesylate and then monitored hematologically every month. Patients are further monitored either cytogenetically every six months until complete cytogenetic response (CCyR) is achieved or molecularly using qRT-PCR, depending on the hematologist's preference. If the cytogenetically monitored patient reaches CCyR, they are monitored biannually using qRT-PCR for the detection of minimal residual disease. In the case of resistance to treatment, a higher dose of imatinib mesylate or a different TKI is administered and the patient is monitored using the same protocol. Contrary to the current recommendations [26],* BCR-ABL* mRNA transcript type is not usually identified. In this study we aimed to identify the frequency of different* BCR-ABL* transcripts in Syrian CML patients and highlight their significance on patient care in order to conclude a better approach to monitoring and treatment.

## 2. Materials and Methods

Patients diagnosed with Ph+ CML at least a year prior and referred to Al-Assad Hospital, Damascus University, for regular monitoring by t(9; 22) qRT-PCR were recruited between January 2012 and November 2014 after obtaining the approval of Damascus University Ethics Committee and informed consents. 3 mL of whole blood was withdrawn on EDTA from each patient. Total RNA was extracted and qRT-PCR was carried out using the High Pure RNA Isolation Kit and the LightCycler-t(9; 22) Quantification Kit (Roche Diagnostics, Germany), respectively, according to the manufacturer's instructions. The resultant RNAs and cDNAs quality was assessed using the NanoDrop ND-1000 Spectrophotometer (NanoDrop Technologies, Inc., USA). Efficient coamplification of* Glucose-6-Phosphate Dehydrogenase* (*G6PDH*) housekeeping gene cDNA was also considered to assess the quality of RNA extracts and the efficiency of cDNA synthesis. cDNA samples that tested positive for* BCR-ABL* transcripts were solely included in our downstream analyses, and those of imatinib-treated patients with* BCR-ABL* transcripts relative levels ≤ 0.1% were categorized as a major molecular response (MMR) group [27].

A home-made method for investigating* BCR-ABL* transcript types included various primer combinations: ABL-a2 with M-BCR, m-BCR, and/or *μ*-BCR [15, 20, 24] (TIB MOLBIOL, Germany; Table 1). The primers sequences were verified using the NCBI nucleotide BLAST (http://blast.ncbi.nlm.nih.gov/). PCRs were optimized on 5 *μ*L of a positive control using 0.1, 0.25, 0.5, or 1 *μ*M of each primer; 1, 2, 3, or 4 mM MgCl_2_; 200 *μ*M dNTPs; 1.25 U AmpliTaq® Gold DNA Polymerase (Applied Biosystems, USA) in a final volume of 50 *μ*L. Thermal cycling was initiated on MasterCycler® Pro S (Eppendorf, Germany) by enzyme activation at 95°C for 5 minutes, followed by 45 cycles of denaturation at 95°C for 15 seconds, annealing at 50°C, 55°C, 56°C, 58°C, or 60°C for 15 seconds, and extension at 72°C for 2:30 minutes, with a final extension at 72°C for 7 minutes. Optimal reaction conditions were then adopted for investigating* BCR-ABL* transcript types in our cDNA samples. PCR products were electrophoresed on a 2.5% agarose gel stained with ethidium bromide.

The cDNA samples were also tested for confirmation of* BCR-ABL* transcript types using the Seeplex® Leukemia* BCR/ABL* research kit (Seegene, Korea) according to the manufacturer's instructions.

Fisher's exact test was used to study independence of* BCR-ABL* transcript type among patient groups using GraphPad QuickCalcs software (http://graphpad.com/quickcalcs/). *P* value < 0.05 was considered significant.

## 3. Results

Twenty-two patients tested positive for* BCR-ABL* transcripts by qRT-PCR, including nine imatinib-treated patients who reached MMR, seven imatinib-treated patients who had not reached MMR, and six noncompliant patients who paused imatinib at least 1.5 year prior to sampling. Imatinib therapy in all treated patients was initiated at least a year before our study.

During the optimization process of the home-made method, PCR products were only amplified using ABL-a2/M-BCR primer combination, on the contrary of ABL-a2/m-BCR, ABL-a2/*μ*-BCR, and ABL-a2/M-BCR/m-BCR/*μ*-BCR primer combinations. However, the optimal reaction conditions of the ABL-a2/M-BCR primer combination (4 mM MgCl_2_, 0.25 *μ*M each primer, and annealing temperature of 55°C) yielded a single faint band in only ten of the 22 samples.

Using the Seeplex Leukemia* BCR/ABL* research kit, a single transcript with a breakpoint in M-*bcr* region, a single transcript with a breakpoint in m-*bcr* region, dual transcripts both with a breakpoint in M-*bcr* region, and dual transcripts one with a breakpoint in M-*bcr* region the other in m-*bcr* region were identified in seventeen, one, two, and one patients, respectively; this amounts to a total of 24 transcripts. PCR failure was encountered in one of 22 samples. The most commonly encountered transcript was b2a2, followed by b3a2, b3a3, and e1a3 present solely in 12 (57.1%), 3 (14.3%), 2 (9.5%), and 1 (4.8%) of the 21 patients, respectively. The three (14.3%) samples containing dual transcripts showed three different combinations of b2a2/b3a2, b3a2/b3a3, and b2a2/e1a3.

Table 2 demonstrates the distribution of* BCR-ABL* transcript types among imatinib-treated patients according to their molecular response as well as noncompliant patients. Being more frequently observed in patients who had not reached MMR, b3a2* BCR-ABL* transcript type was apparently associated with warning molecular response to imatinib treatment (*P* = 0.047). On the contrary, b2a2, b3a3, and e1a3* BCR-ABL* transcript types were not significantly associated with the molecular response to imatinib treatment (*P* > 0.05).

## 4. Discussion

*BCR-ABL* mRNA transcript type detection by research kits is relatively expensive and may lack the ability to detect unknown transcripts. This led us to investigate a more economic and comprehensive home-made conventional PCR method for integration into routine monitoring protocols. Types of the ten detected transcripts using our home-made method were consistent with the research kit results; however, PCR failure was frequently encountered losing 58% of the expected transcripts in our known positive samples. Faint bands of M-*bcr* breakpoint reaction products as well as failure of both m-*bcr* breakpoint reaction and M-*bcr*/m-*bcr*/*μ*-*bcr* multiplex reaction could be attributed to primer depletion from the reaction mix, evidenced by the appearance of primer-dimer bands. These disappointing findings come despite our careful selection and thorough optimization of published primers. Albeit previously described by several research teams [15, 20, 24] who adapted them from an earlier work [2], suggesting reproducibility, these primers proved otherwise. Since published methods may not be reliably reproducible, empirical verification is essential before integration into routine use.

Results obtained using the research kit were consistent with the qRT-PCR in terms of* BCR-ABL* transcript positivity, with PCR failure showing only in one of 22 samples. Albeit quantifiable by qRT-PCR, low-level transcript types might have been missed due to the limited sensitivity of conventional PCR. However, the kit was discriminative showing inter- and intrasample variations in transcript types. Obviously, b2a2* BCR-ABL* transcript accounts for the two-thirds of majority of all transcripts detected in Syrian CML patients, while one-fourth of the patients expressed the b3a2 transcript either solely or in addition to another transcript. This coincides with previous prevalence estimates in other countries where b2a2 transcript was the most frequent [12–15] but contradicts other reports where b3a2 transcript was the most prevalent [16–24]. It seems that the frequency of each* BCR-ABL* transcript, whether common or rare, and the percentage of patients showing coexpression of different transcripts varies widely between different areas.

Despite our small sample size, this study evidenced an apparent association between the b3a2* BCR-ABL* transcript and a poor outcome. This concurs with previous studies showing b3a2 transcript associated with a worse prognosis than b2a2 transcript. One-quarter of our patients with the b3a2 transcript should hence be more rigorously and frequently monitored, where second line TKIs might be recommended to overcome potential resistance. Albeit not significantly associated with the MMR to imatinib treatment each, supposedly attributable to small sample size, b2a2, b3a3, and e1a3 transcripts were collectively associated with MMR. This is underlined by previously reported evidence of better prognosis associated with such transcripts [7, 25, 27].

One-fourth of our patients with rare b3a3 and e1a3 transcripts represent a challenge at the level of molecular diagnosis and monitoring since those transcripts may be undetectable by many commercially available and routinely utilized qRT-PCR assays [25]. The research kit we used for qRT-PCR was sufficient in quantifying all detected transcripts, but other products may not have this versatility. Thus, a diagnostic approach that can only detect the two most common transcripts b2a2 and b3a2 will miss quantifying one-fifth of the transcripts present in our patients, producing misleading results. Hence, it might be imperative to identify the type of transcript in each CML patient and choose an appropriate monitoring protocol that is known to detect the transcript type identified in order to avoid subsequent false-negative qRT-PCR results.

## 5. Conclusion

In conclusion, we recommend identifying the* BCR-ABL* transcript type in every CML patient at diagnosis along with the cytogenetic study, using a reliably reproducible and empirically verified method. We propose either a commercially available kit like the one used in this study or a more successful home-made PCR method utilizing different primers. Linking the detected transcript with the patient's clinical findings, possible resistance to first line treatments, and appropriate monitoring methods eventually might help establish more efficient treatment protocols based on the prevalent disease phenotypes in our population.

## Figures and Tables

**Table 1:** Oligonucleotide primers sequences used in the home-made PCR method.

Primer name	Primer sequence 5′-3′
ABL-a2 (reverse)	5′-TGT TGA CTG GCG TGA TGT AGT TGC TTG G-3′
M-BCR (forward)	5′-ACA GMA TTC CGC TGA CCA TCA ATA AG-3′
m-BCR (forward)	5′-ACC GCA TGT TCC GGG ACA AAA G-3′
*μ*-BCR (forward)	5′-GAA GAA GTG TTT CAG AAG CTT CTC CC-3′

**Table 2:** Frequency of *BCR-ABL* transcript types among CML patient groups (*n* = 21)^†^.

Patient group	*BCR-ABL* transcript types			
	b2a2	b3a2	b3a3	e1a3
*Imatinib-treated patients (n* = 15)	*P* = 0.454^*∗*^	*P* = 0.047^*∗*^	*P* = 0.657^*∗*^	*P* = 0.342^*∗*^
*BCR-ABL* ≤ 0.1% (*n* = 9)	6 of 10 (60%)	1 of 10 (10%)	1 of 10 (10%)	2 of 10 (20%)
*BCR-ABL* > 0.1% (*n* = 6)	3 of 8 (37.5%)	4 of 8 (50%)	1 of 8 (12.5%)	0 of 8 (0%)

*Noncompliant patients (n* = 6)	5 of 6	0 of 6	1 of 6	0 of 6

^†^Dual transcripts were detected in three patients, which explains reporting 24 transcripts in 21 patients.

^*∗*^Independence of *BCR-ABL* transcript types among imatinib-treated patients according to their molecular response was analyzed by Fisher's exact test. *P* value < 0.05 was considered significant.
